# The Anti- and Pro-Tumorigenic Role of Microbiota and Its Role in Anticancer Therapeutic Strategies

**DOI:** 10.3390/cancers15010190

**Published:** 2022-12-28

**Authors:** Giulia Greco, Sabrina Donati Zeppa, Deborah Agostini, Giuseppe Attisani, Claudio Stefanelli, Fabio Ferrini, Piero Sestili, Carmela Fimognari

**Affiliations:** 1 Department of Chemistry “Giacomo Ciamician”, University of Bologna, 40126 Bologna, Italy; 2 Department of Biomolecular Sciences, University of Urbino Carlo Bo, 61029 Urbino, Italy; 3 Department for Life Quality Studies, University of Bologna, 47921 Rimini, Italy

**Keywords:** microbiota, cancer, probiotics, genotoxicity, cancer therapy

## Abstract

**Simple Summary:**

Human gut is colonized by a wide variety of microorganisms, which collectively form the so-called microbiota. A healthy microbiota interacts with our organism as a symbiont positively participating in the regulation of many physiological activities of the host, detoxification of xenobiotics, and prevention of the development of specific pathologies. However, various conditions may change the status of microbiota from “healthy” to “unhealthy”; an unhealthy status may negatively impact the host health contributing to the onset of maladies. Cancer is among those that microbiota can prevent or promote according to its conditions. The knowledge of the situations and of the mechanisms underlying this bifaceted role of microbiota is of great importance in cancer prevention, epidemiology, diagnosis, prognosis, and therapy. Here we will illustrate and discuss how and when microbiota interferes with development and progression of human cancer, with a particular emphasis on colorectal cancer.

**Abstract:**

Human gut microbiota physiologically and actively participates as a symbiont to a wide number of fundamental biological processes, such as absorption and metabolism of nutrients, regulation of immune response and inflammation; gut microbiota plays also an antitumor role. However, dysbiosis, resulting from a number of different situations—dysmicrobism, infections, drug intake, age, diet—as well as from their multiple combinations, may lead to tumorigenesis and is associated with approximately 20% of all cancers. In a diagnostic, prognostic, therapeutic, and epidemiological perspective, it is clear that the bifaceted role of microbiota needs to be thoroughly studied and better understood. Here, we discuss the anti- and pro-tumorigenic potential of gut and other microbiota districts along with the causes that may change commensal bacteria from friend to foes.

## 1. Introduction

Cancer is the second leading cause of death in the world. Recently, the role of microorganisms in cancer development has been identified. In particular, the gut microbiota, a very complex community of commensal microorganisms including bacteria, Archaea, fungi, protists, and viruses, has emerged as a critical player in the modulation of cancer development. Indeed, the commensal microbiota induces immunological and cellular pathways to prevent cancer and activates inflammasomes, which preserve the host’s cellular and gut integrity [1]. Accordingly, the amelioration of microbiota through the intake of probiotics is recognized as a chemopreventive intervention favoring improvement of the intestinal barrier, bacterial translocation, and maintenance of gut microbiota homeostasis and composition; healthy microbiota also exhibits detoxifying activity against chemical genotoxic agents. The perturbation of microbiota’s homeostasis may turn microbiota into a tumorigenic player, especially with regard—but not limited to—colorectal cancer, which represents a major worldwide cause of cancer-related deaths [2]. Some gut microorganisms may actually be involved in progenotoxic and pro-carcinogenic processes through different mechanisms, including the production of bacterial-derived genotoxins, microbial-derived metabolites, modulation of host defenses and inflammation pathways, oxidative stress induction, anti-oxidative defense imbalance, and modulation of tumor microenvironment [3]. In analogy with gut microbiota, other compartments’ microflora—i.e., skin and pulmonary—may also play a bifaceted role in tumor formation and progression.

Another relevant aspect of gut microorganisms is their ability to modulate the efficacy and the toxic burden of antitumor chemotherapeutic drugs [4] and, most notably, cancer immunotherapy [5,6,7,8,9]. 

In this review, we highlight the relationships between the gut microbiota and other compartments’ microbiota and cancer. Moreover, we present the possible mechanisms that may be used by the microbiota to modulate the carcinogenetic process in a bilateral way, which may lead to cancer development or may be used as a strategy for cancer treatment and prevention.

## 2. Antigenotoxic Effects of Gut Microbiota

A genotoxin is a chemical or physical agent that modifies DNA or modifies the cells’ ability to control DNA structure and content. Damage to DNA may or not lead to a permanent modification in its content or structure, depending on several factors, including DNA repair enzymes, detoxification, cell death, and antioxidant systems. Genotoxicity occurring in germ cells may lead to a heritable altered trait, while in somatic cells may result in adverse outcomes such as cancer, cardiovascular diseases, neurodegenerative diseases, and developmental toxicity [10].

Spontaneously occurring DNA lesions are common in the DNA at any time, which are usually counteracted by repairing systems. However, there are many compounds able to increase the number of DNA lesions to the level that leads to tumorigenesis. Many of them lack a threshold of dose or act at such a low dose and exposure levels that they have little practical impact in guaranteeing safe exposure. As such, testing for potential genotoxicity is extremely important for chemicals and products risk assessment and predicting whether their exposure can be safely managed [11].

There are several in vitro and in vivo genotoxicity tests which are able to detect the different types of genotoxic effect, such as point mutations (i.e., base-pair substitutions and frame-shift mutations), DNA single- or double-strand breaks, and loss or gain of whole chromosomes. Moreover, there are substances that act as indirect genotoxins. In particular, they can produce reactive oxygen species, deplete cellular antioxidant systems, or inhibit topoisomerases or kinases and cause point mutations, DNA breaks, or alteration of chromosomes’ number. 

The presence of substances capable of causing DNA damage in the environment as well as in certain foods poses serious risks to human health. Exposure to several xenobiotics, such as polycyclic aromatic hydrocarbons, aflatoxins, and heterocyclic aromatic amines, is associated with an increased risk of developing different cancer types [12]. Clearly, the first intervention strategy would be to minimize the possible exposure to these substances. Although stringent rules can be put in place to regulate their presence either in food or at an environmental level, it is not possible to eliminate the exposure to genotoxic substances. Thus, the search for compounds capable of counteracting the DNA damage of food and environmental xenobiotics turns out to be extremely important [13,14,15].

Antigenotoxic agents are classified into desmutagens or bioantimutagens, based on their mechanisms of action. The former class acts by preventing DNA damage, the latter by repairing it. Desmutagens can act at both intra- and extra-cellular levels. They can prevent DNA damage through the following: 1. the inhibition of the toxic agent’s entry into the cell; 2. the irreversible binding to the genotoxic agent itself; 3. the chemical inactivation of genotoxic compounds through modulation of phase 1 and 2 enzymes; 4. the inhibition of oxidative stress, if the damage is due to oxidative stress, by acting as antioxidants. Bioantimutagens act instead at the intracellular level and prevent the replication of damaged DNA and/or promote its repair [16]. 

In recent years, numerous studies focused on the possible antigenotoxic activity of specific bacteria or yeasts, defined as probiotics. A series of assays were employed to assess probiotics’ antigenotoxicity. They measure 1. DNA strand breakage such as Comet assay (single-cell gel electrophoresis) or SOS chromotest; 2. expression of proteins involved in the bacterial SOS repair system; 3. reverse mutations at selected loci of several bacterial strains, such as the Ames test; 4. chromosomal damage such as the micronucleus assay [17]. However, some gut microorganisms may be involved in progenotoxic and pro-carcinogenic processes through different mechanisms, including the production of bacterial-derived genotoxins, microbial-derived metabolites, modulation of host defenses and inflammation pathways, oxidative stress induction, anti-oxidative defense imbalance, and modulation of tumor microenvironment [3]. Another relevant aspect of gut microorganisms is their ability to modulate the efficacy and the toxic burden of antitumor chemotherapeutic drugs.

The antigenotoxic activity of probiotics has been widely investigated against food and environmental genotoxic contaminants such as 4-nitroquinoline 1-oxide (4-NQO) [18], 2-nitrofluorene, aflatoxins (AFs), heterocyclic aromatic amines (HAAs), N-nitrosamines [19], and polycyclic aromatic hydrocarbons (PAHs) [20]. In the next paragraphs we will present and discuss the antigenotoxic effect of probiotics toward food and environmental genotoxins. Experimental details are presented in Table 1 and Table 2, which report in vitro and in vivo studies, respectively. 

### 2.1. Antigenotoxic Effects of Probiotics toward Heterocyclic Aromatic Amines (HAAs) and N-Nitrosamines

IARC included HAAs such as 2-amino-3-methylimidazo[4,5-f]quinoline into class 2A (i.e., probably carcinogenic to humans), and 2-amino-1-methyl-6-phenylimidazo(4,5-b)pyridine (PhiP), 2-amino-3,4-dimethylimidazo(4,5-f)quinoline, 2-amino-3,4-dimethylomidazo[4,5-f]quinoline (MeIQ), 2-amino-3,8-dimethylimidazo[4,5-f]quinoxaline (MeIQx), and 3-amino-1,4-dimethyl-5H-pyrido[4,3-b]indole (Trp-P-1) in class 2B (i.e., possibly carcinogenic to humans). 

Different Lactic Acid Bacteria (LAB), such as lactobacilli (*Lb*.) and bidifobacteria, exhibited antigenotoxic effects toward various HAAs (Table 1 and Table 2). Using the Comet assay, it has been found that *Lb. casei* 0919 and *Lb. rhamnosus* 0908 reduced the DNA damage induced by IQ and PhIP (50 μg/mL) by up to 80% on Caco-2 cells [39]. All the tested bacterial strains efficiently bound HAAs [39], and *Lb. rhamnosus* 231 exhibited the same effects toward MeIQx [22]. These results indicate that the antigenotoxic effect of LAB may rely on their ability to bind genotoxins. LAB, as other microorganisms, can synthesize exopolysaccharides (EPSs). They are secreted as either capsular EPSs, where EPS are tightly attached to cell surface, or as slime EPSs released extracellularly as free polymers [53]. Tsuda et al. correlated the ability of antigenotoxic activity of probiotics to the presence of EPSs [36]. They tested an FM prepared with two different strains of *Lb. plantarum*: the parental strain (301102) and a mutant strain (301102S) capable of producing capsular EPSs after its exposure to the mutagens acridine orange and novobiocin. Investigating the antimutagenic activity of FM containing 301102 and 301102S strains as well as lyophilized EPSs alone, they found that FM prepared with the parental strain did not exert any antimutagenic effect, whilst both FM with mutant strain and lyophilized EPSs alone strongly reduced the mutagenic effect of Trp-P-1 (0.1 mg/mL). Thus, the antimutagenic activity was attributed to the presence of EPSs and their ability to bind the mutagen. Indeed, among capsular cells (i.e., cells with EPSs on cell surface), cells, and peptidoglycans of parental and mutant strains, only capsular cells of 301102S strain efficiently bound Trp-P-1 by more than 50%. These results indicate that the presence of EPSs is essential for the binding activity. Although to a minor extent, capsular cells of strain 301102S bound also 2-amino-6-methyldipyrido imidazole (Glu-P-1), MeIQ, and N-methyl-N′-nitro-N-nitrosoguanidine (MNNG) [36]. The binding activity of capsular cells to Trp-P-1 was further investigated, demonstrating that it was significantly affected by pH, MgCl2, and sodium dodecyl sulfate (SDS). The maximum % of binding was reported at pH 8.0, while MgCl2 and SDS decreased and entirely inhibited the binding to Trp-P-1, respectively. These findings suggest the involvement of cation exchange and the formation of hydrophobic bounds as binding mechanisms of EPSs [36]. In contrast, the addition of oxgall up to 0.4%, which simulates a bile content of 4%, showed no influence on the binding ability of EPSs, suggesting that EPSs likely retain their binding activity in the digestive tract [36].

Other mechanisms are involved in the antigenotoxic activity of probiotics toward HAAs. *Lb. rhamnosus* 231, after binding to MeIQx, bio-transformed and detoxified it, as observed by the changes in the UV spectrum observed after the co-incubation with the probiotic [22]. *Lb. casei* DN 114001 lowered the concentration of IQ, MeIQx and PhIP (all tested at 25 μg/mL) detected in the culture medium. The composition of culture medium influenced the observed decrease in HAAs levels. In general, a greater reduction was observed using bacterial cells grown in MRS (De Man, Rogosa, Sharpe) (i.e., growing cells) than in phosphate buffers (i.e., non-growing cells). This difference could be attributed to the different pH. Following incubation, the pH of the phosphate buffer remains constant during cultivation (6.2–6.3), while that of the MRS broth becomes more acidic due to bacterial fermentation and subsequent lactic acid production [21]. However, the modification of MRS broth (i.e., with a reduced percentage of carbon and nitrogen) also reduced the concentration of HAAs. In particular, the compounds were partially (IQ) or completely (MelQx) desorbed to the modified MRS medium. These results confirm that both growing and non-growing *Lb. casei* DN 114001 cells can metabolize the genotoxins [21]. Antigenotoxicity was assessed by Comet assay. *Lb. casei* DN 114001 reduced the genotoxicity of IQ, MeIQx and PhIP (all tested at 25 μg/mL) differently depending on culture medium and the tested genotoxin. In MRS broth, bacterial cells reduced the genotoxicity of IQ by 99%, but there was no statistically significant inhibition against MeIQx and PhiP despite their reduced concentrations (Table 1). As stated by the authors, this was probably due to the release of genotoxic metabolites. After cultivation in phosphate buffer, *Lb. casei* DN 114001 reduced the genotoxicity of all three HAAs, including MeIQx whose concentration in the medium had not been reduced by the bacterial cells. This confirms that different pH leads to different metabolism of HAAs with the production of genotoxic metabolites, in the case of MRS broth, or less or non-genotoxic derivatives, in the case of phosphate buffer [21]. In conclusion, the antigenotoxic effect of *Lb. casei* DN 114001 was mainly due to the ability of bacterial cells to adsorb and metabolize HAAs, rather than their ability to bind them [21]. Similarly, *Lb. casei* 0919 inhibited IQ genotoxicity of about 80%, but bound only the 2% of the genotoxin [39].

A study investigated the ability of different Lb. strains (*Lb. acidophilus* J76, *Lb. casei* 5H10, *Lb. delbrueckii* J87 subsp. *bulgaricus*, *Lb. fermentum*, *Lb. plantarum* J25, *Lb. rhamnosus* J54) to inhibit DNA damage produced by aqueous extracts of six heated cooking oils (extra-virgin olive, peanut, sunflower, soybean, corn, and different seeds oils), which might better mimic human exposure to HAAs through the diet [42]. SOS chromotest unveiled that all probiotic strains inhibited at least the 50% of the genotoxic effect of the different fried oils (Table 1). The most active were *Lb. acidophilus* J76, *Lb. casei* 5H10, *Lb. delbrueckii* J87 subsp. *bulgaricus*, and *Lb. rhamnosus* J54 with a median inhibition of 78%, and *Lb. delbrueckii* J87 subsp. *bulgaricus* that inhibited the 88% of genotoxicity. In contrast, *Lb. fermentum* and *Lb. plantarum* J25 were the most ineffective. Differences were found not only within bacterial strains, but also among the different types of oil examined: the genotoxic activity of corn, different seeds, and extra-virgin olive oil was greatly suppressed compared to that of peanut oil. These results reflect the different genotoxicity exhibited by the oils, with corn, other seeds, and peanut oil being the most genotoxic and peanut oil being the least. Additionally, the various chemical composition of the oils, leading to the production of different genotoxic substances during the frying process, may also influence the antigenotoxic effect of probiotic strains [42]. Of note, heat-treated bacterial cells did not show any protective activity [42]. Probiotics’ ability to inhibit HAAs genotoxicity was also confirmed in vivo. In male CD-1 mice, administration of *Lb. rhamnosus* IMC501 (10^9^ cells/mL, 10 mL/kg b.w.) for 10 days prior to the feeding with PhIP (100 mg/kg b.w.), inhibited the genotoxicity of PhIP of about 70%, as demonstrated by the Comet assay performed on colonic cells [44]. In another study, Horie et al. tested the ability of a probiotic mixture containing *Streptococcus faecalis* (10^8^ cfu/g), *Clostridium bothrium* (10^7^ cfu/g) and *Bacillus mesentericus* (10^6^ cfu/g) to inhibit the genotoxicity of the aromatic amine AAC (2-amino-alpha-carboline) in a human-flora-associated (HFA) mice model, using germ-free NMRI mice. The probiotic mixture was administered for 2 weeks before the oral administration of AAC (40 mg/kg b.w.) once a day for 3 days. Results showed a 35% reduction of DNA adduct formation induced by AAC in the colonic epithelium of HFA mice (Table 2) [45].

Some food products contain N-nitrosamines, as N-nitroso-dimethylamine (NMDA), which are primarily formed by the high-temperature reaction of nitrite with nitrosatable amines in meat, fish, and other foodstuffs [19]. N-nitrosamines are also present in the environment, formed by combustion processes, as well as in pesticides, rubber, beer, cosmetics, as a result of technical processes [19]. According to the IARC, ingested nitrate or nitrite in conditions resulting in endogenous nitrosation is probably carcinogenic to humans (Group 2A). Ames test was used to assess the antigenotoxic activity of forty strains isolated from an FM (Table 1). All LAB at least halved the mutagenic activity of NDEA (60 μM). The most effective among them were *Streptococcus lactis* subsp. *diacetylactis* (R-63), *Streptococcus cremoris* (R-48), and *Leuconostoc paramesenteroides* (R-62 and R-8), which inhibited 99.14%, 98.71%, 98.19%, and 86.17% of revertant colonies, respectively [31]. Considering the high antigenotoxic activity, those strains were also investigated against three other volatile N-nitrosamines: NMDA, N-nitroso-pyrrolidine (NPYR), and N-nitroso-piperidine (NPIP). The results suggested that lyophilized LAB (5 and 7 mg) strongly reduced the mutagenicity of NMDA (at least 50%) but marginally that of NPYR and NPIP (less than 15%) [31]. Several factors influence the potency of nitrosamines: 1. their cyclic or acyclic structure; 2. the size of the ring, if any; 3. the presence of only methyl or ethyl groups. Acyclic nitrosamines are more toxic than cyclic ones, and nitrosamines that possess only methyl groups, such as NMDA, or ethyl groups, such as NDEA, are extremely potent in terms of genotoxicity [54]. Probably, these differences can also be reflected in the antigenotoxic effect since LAB were remarkably effective against NMDA and NDEA, but very poorly against the cyclic NPYR and NPIP, characterized by the presence a pyrrole (NPYR) or a piperidine ring (NPIP). An in-depth investigation was conducted by Nowak et al., who examined the ability of four LAB strains to detoxify and thus decrease NMDA levels [30]. Bacterial strains decreased NMDA levels in a strain-dependent manner and differently depending on the culture medium used and incubation time. After cultivation for 24 h in MRS broth, all strains reduced NMDA levels (from 20% to 46%) starting from an initial low NMDA concentration (2 μg/mL). Increasing the initial concentration of NMDA to 20 μg/mL, only *Lb. rhamnosus* 0908 and *Lb. casei* DN 114001 reduced of about 30% the genotoxin’s levels, while the other bacterial strains did not produce any drop. In modified MRS broth, a decrease was observed during the logarithmic and death phase of growth, while in the stationary phase NMDA concentration increased to the initial level. These results show that even inactive bacterial cells detoxified NMDA. *Lb. rhamnosus* 0908 and *Lb. casei* 0919 were unable to reduce the genotoxin’s levels [30]. After 168 h incubation in phosphate buffer, the drop of NMDA concentration in supernatants was lower than in MRS broth. The highest decrease was observed following incubation of NMDA at starting concentration of 20 μg/mL with *Lb. brevis* 0945 (41.5%), while *Lb. rhamnosus* 0900 and 0908 did not produce any drop [30]. To analyze the mechanisms underlying this decrease, bacterial cells were incubated for 168 h in MRS broth with NMDA (10 μg/mL) and then the concentration in membrane and intracellular extracts as well as supernatants was quantified. NMDA was found in both membrane extracts (0.15–0.26 μg/mL) and supernatants (6.09–8.94 μg/mL). Thus, both genotoxin’s metabolism and adsorption by bacterial cells may be involved in the protective effects of the probiotics tested [30]. Of note, intracellular extracts reduced NMDA levels, particularly that of *Lb. rhamnosus* 0908. This means that NMDA could also be eliminated by enzymatic reactions occurring in intracellular extracts [30]. In the same study, Nowak et al. also investigated the ability of probiotic bacterial strains to inhibit NMDA genotoxicity using the Comet assay (Table 1). Antigenotoxic effect was greater when cells were incubated with NMDA (10 μg/mL) for 168 h in phosphate buffer rather than for 24 h in MRS broth. In the latter case, the antigenotoxic activity of *Lb. rhamnosus* 0908 and *Lb. brevis* 0945 was the most effective, reducing 32.0% and 49.8% of NMDA genotoxicity, being correlated to their greater reduction in NMDA concentration. The other strains slightly inhibited the genotoxic effect of NMDA (from 2.4% to 13.1%). On the other hand, bacterial cells cultured in phosphate buffer for 168 h, except for *Lb. casei* DN 114001, caused a significant inhibition of the nitrosamine’s genotoxicity. The highest effect was recorded for *Lb. casei* 0919, which reduced 71.3% of NMDA genotoxicity. The different protective effect could be ascribed to a more acidic pH of MRS broth than a neutral pH of phosphate buffer [30]. As previously described, similar findings were observed for the antigenotoxic effect *Lb. casei* DN 114001 towards HAAs [21].

### 2.2. Antigenotoxic Effects of Probiotics toward Polycyclic Aromatic Hydrocarbons (PAHs)

PAHs are environmental pollutants resulting from uncompleted combustion of organic matter [55]. In food, heat treatment for cooking or processing generates PAHs that could be taken in through the ingestion of certain foods as oils, grains, and vegetables [56]. PAHs possess mutagenic, carcinogenic, teratogenic, and immunotoxic effects [57]. Benzo[a]pyrene (BaP), one of the most toxic PAHs, is metabolized by the CYP450 family enzymes resulting in the metabolite 7,8-dihydroxy-9,10-epoxy-BaP, capable of binding DNA and generating covalent adducts. From 2012, BaP has been included by the IARC in the Group 1, i.e., carcinogenic to humans. 

Janosch et al. examined the antimutagenic properties of *E. coli* nissle 917 towards BaP (100 μg/mL, 396 μM) using Ames test (Table 1), showing that viable, heat- and UV-treated bacterial cells decreased BaP mutagenicity of about four-fold, while sonication totally prevented any protective effect. The bacteria-free supernatant also lacked antimutagenic effects [43]. A possible mechanism by which *E. coli* could neutralize BaP mutagenicity was the absorption of the mutagen and the subsequent release of glutathione from bacterial cells. Moreover, as glutathione is resistant to heat, heat-treated cells retained the protective effect towards BaP mutagenicity [43]. Corroborating the hypothesis that heat-treatment does not influence probiotics’ antigenotoxic effect, Shoukat et al. reported that both viable and heat-killed bacterial cells of three *Bifidobacterium* strains bound to BaP to the same extent [58]. In contrast, the antimutagenic properties of *E. coli*, as well as other bacterial cells, towards 4-NQO mainly relies upon the ability of viable bacterial cells to rapidly degrade 4-NQO into non-toxic metabolites. This different detoxifying mechanism could explain why heat-killed cells of *E. coli* did not exert any protective effect toward 4-NQO [43].

Besides binding and absorption, also the increment of the antioxidant defenses largely contributes to minimize BaP-induced DNA damage. Co-incubation of BaP (50 μM) with *Bifidobacterium animalis* subsp. *lactis* BI-04 (about 5 × 10^8^ cfu/mL) markedly increased SOD activity, intracellular levels of glutathione, and the expression of antioxidant and metabolism-related enzymes in Caco-2 cells and led to a decrease in BaP-induced DNA damage, as reported using the Comet assay [40]. Similarly, the co-administration of *Lb. plantarum* CICC 23121 (5 × 10^10^ cfu/mL) and BaP (50 mg/kg) to clean-grade Kunming male and female mice increased hepatic total antioxidant capacity, and catalase and SOD activity as well as the fecal excretion of BaP, thus reducing the extent of DNA damage in rat peripheral blood cells [46]. Thus, the ability to increase intracellular antioxidant systems seems to be involved also in the probiotics’ antigenotoxicity exhibited in vivo.

**Figure 1:**
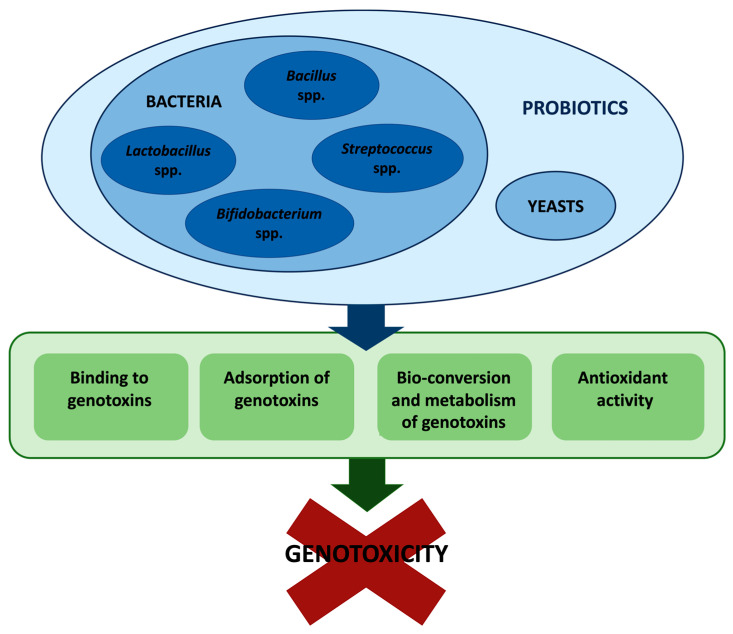
Overview of the mechanisms involved in probiotics’ antigenotoxic activity.

In conclusion, many in vitro and in vivo studies demonstrated that probiotics exhibit a marked antigenotoxic activity toward various environmental- and food-related genotoxins. Multiple mechanisms are involved in the antigenotoxic activity of probiotics. Primarily, probiotics act as desmutagens. Indeed, they have been found 1. to bind and detoxify genotoxic agents, thus inhibiting or lessening the formation of the ultimate genotoxins; 2. to adsorb genotoxins; 3. to enhance intracellular antioxidant defenses (Figure 1). Because of their ability to repair DNA damage, some probiotic strains act also as bio-antimutagens [47,59], even though further studies would be needed to explore and understand the mechanisms behind it.

Almost all studies found a strain-dependent antigenotoxic effect. Furthermore, the same bacterial strains showed varying degrees of geno-protective activity depending on the tested genotoxin. This is most likely due to the increased toxicity of one genotoxin over another [30], or depending on whether the genotoxin is metabolized, resulting in non-toxic or genotoxic metabolites [21]. In addition, also pH and culture medium could affect the geno-protective activity of probiotics [21,30]. Conflicting results exist regarding the ability of inactivated probiotic bacterial cells to exert antigenotoxic activity. Different studies showed that when genotoxins were co-incubated with heat-treated bacterial cells, the antigenotoxic effect was significantly decreased or totally deleted [25,26,33,41]. In other studies, instead, heat-treated bacterial cells retained their antigenotoxic activity, although to a lesser extent than viable cells [27,38,43]. This different behavior may be due not just to the diverse bacterial strains and species, but mainly to the underlying mechanism of the probiotic’s antigenotoxic effect. In fact, it seems that when the main protective mechanism is genotoxin’s metabolism, as in case of 4-NQO, only viable bacterial cells exhibit geno-protective effects, because inactivated cells are likely no longer able to metabolize the genotoxin. In contrast, if genotoxin’s binding or adsorption is the principal protective mechanism, as in the case of AFs and BaP, the antigenotoxic activity appears to be retained even by inactivated bacterial cells. 

## 3. Pro-carcinogenic Effects of Gut Microbiota

Although the role of gut microbiota in carcinogenesis is generally associated to its preventive role, an emerging aspect of research points to the pro-carcinogenic effect that commensal and/or pathogenic bacteria can exert under certain circumstances, possibly associated to an increased risk of colorectal cancer (CRC). With ca. 1.9 million worldwide new instances per year, CRC is the second and third common cancer in females and males, respectively, and the second leading cause of cancer deaths. 55% of CRC occur in developed countries where, despite the significant improvement in the treatment options, it poses an increasing burden for public health. Many risk factors—the most common being diet habits, smoke, alcohol consumption, sedentary life and age—concur to the development of CRC. Due to its high sociosanitary impact, the identification of new risk factors is of great importance for both preventive and treatment purposes.

In this light, microbiota is increasingly being regarded not only as an antigenotoxic player, but also, under specific circumstances, as a CRC risk factor. Some pathogenic strains have been associated to gastrointestinal tumorigenesis, such as *Streptococcus bovis*, *Fusobacterium nucleatum*, and *Enterococcus faecalis* [60,61,62], but also commensal strains, may play a role in CRC. Human gut microbes may promote, mitigate, or have no direct effect on carcinogenesis. Microbes such as *Helicobacter pylori*, *Fusobacterium. nucleatum*, enterotoxigenic *Bacteroides fragilis*, and *Peptostreptococcus anaerobius* have been reported to amplify cancer development and progression via toxins production and activation of pro-carcinogenic signaling pathways. 

The pro-carcinogenic impact of microbiota has been proposed to involve three main mechanisms:

(1) alterations of microbiota composition leading to immune evasion, chronic inflammation, and alteration of proliferative responses, which in turn may promote carcinogenesis;
(2) generation of genotoxic metabolites through the bacterial activity of biotransformation;
(3) direct production of bacterial genotoxins.

Recently, the main mechanisms driven by gut microorganisms, at different stages of cancer development have been described in detail [63].

Microbiota exerts a protective role in intestinal lumen integrity by competing with bacterial pathogens and preventing their growth, promoting mucin production and epithelial cell turnover. Mechanical alteration of the barrier function, which can result from microbial dysbiosis [64], facilitates the access of bacteria to the underlying connective tissue, causing local inflammation. The persistence and chronicization of inflammation—which may not only depend on the loss of barrier function-activate immune system reactivity via pattern recognition receptors [65] such as Toll-like (TLR) and NOD-like receptors [66]—alter the normal cell death/proliferation balance, generate DNA-damaging reactive oxygen (ROS) and reactive nitrogen species (RNS), and finally facilitate tumorigenesis. Inflammation-dependent activation of nuclear factor k-light-chain-enhancer of activated B cells (NF-kB) and Wnt-β catenin signaling has been reported to foster reprogramming and dedifferentiation of epithelial cells into tumorigenic stem-cell-like cells [67,68].

Increased barrier permeability not only creates a dysbiotic environment, but may also pave the way to bacterial pathogens infiltration and colonization. The combination of both events further sustains the tumorigenic loop. In this context, an increasing role is being attributed to pathobionts [69] such as *Enterococcus faecalis*, *Fusobacterium nucleatus*, adherent invasive *E. coli*.

*E. faecalis*, a commensal pathobiont normally present in human microbiota, has been reported to cause macrophage polarization to M1 phenotype in interleukin-10 (IL-10) deficient mice [70]. Polarized macrophages secrete not only proinflammatory cytokines, but also ROS and RNS, and clastogens such as 4-hydroxy-nonenal, which can foster inflammation and directly damage the DNA of intestinal epithelial cells [71]. In primary colon epithelial cells and colons from IL-10 knockout mice, *E. faecalis*-polarized macrophages were also found to activate β-catenin signaling and induce the pluripotent transcription factors c-Myc, Klf4, Oct4, and Sox2 and cancer stem cells markers [72]. This microbiota-driven mechanism, known as bystander effect, has been proposed to cause endogenous chromosomal instability, cellular transformation and initiation of CRC [62]. Although the microbiome-driven bystander effect has not been demonstrated in humans, it is important considering that specific commensals have the potential to trigger pro-carcinogenic events under specific favoring conditions through complex mechanisms.

*Fusobacterium* species, in particular *F. nucleatum*, seem to induce tumorigenesis and/or to be associated with an increase in CRC. In particular, *F. nucleatum* has been involved in the formation of a proinflammatory microenvironment in the proximity of the tumor and facilitating its progression [73]. Different studies have demonstrated an enrichment of *F. nucleatum* in human colorectal adenomas and carcinomas compared to adjacent normal tissue [74]. Increased tissue *F. nucleatum* DNA was found to correlate with advanced CRC phases and shorter survival [75]. The relative abundance of *F. nucleatum* in patients diagnosed with CRC is being actively investigated to unravel its diagnostic, therapeutic, and prognostic significance [76,77].

Microbiota may participate in determining the pro- or anti-carcinogenic effect of diet habits. For example, the consumption of red meat favors the imbalance toward commensal proteolytic microbes associated to an increased generation of genotoxic metabolites accumulated upon proteolytic fermentation, such as N-nitroso compounds, ROS, RNS, heterocyclic amines, and p-cresol. High levels of p-cresol can be found in human feces (ca. 0.5 mM), but due to the high urinary excretion of its metabolite p-cresol sulfate, even higher levels are likely to accumulate in the colon. Notably, millimolar concentrations of p-cresol are capable of damaging the DNA from human colonic epithelial adenoma Caco-2 and HT29 cells. Lower p-cresol concentrations resulted in a decreased DNA damaging load, but induced a significant mitogenic response in Caco-2 and HT29 cells, which suggests that under these conditions p-cresol may also act as a tumor promoter [78]. To date, although studies failed to find a correlation between p-cresol sulfate urinary levels and CRC, the data from the above mechanistic study suggest that p-cresol sulfate urinary levels may represent a valid biomarker for CRC risk [78].

Commensal sulfate reducing bacteria are mainly represented by Deltaproteobacteria -Desulfobacterales, Desulfovibrionales and Syntrophobacterales [48] followed by the second largest group including the Firmicutes genera, Desulfotomaculum, Desulfosporomusa and Desulfosporosinus [79]. These anaerobic bacteria extract energy from organic molecules or hydrogen oxidation coupled with sulfate reduction to H_2_S. H_2_S-via free radicals’ formation-damages nuclear DNA from colonic epithelial cells at concentrations ≥ 1 μM, possibly leading to genomic instability and/or the cumulative mutations found in CRC [80].

Some Clostridia—*Clostridium scindens*, *C. hiranonis*, *C. hylemonae* (C. cluster XVIa), and *C. sordellii* (C. cluster XI)—are involved in the 7-dehydroxylation of primary bile acids to form the secondary bile acids deoxycholic acid (DCA) and lithocholic acid (LCA) [81]. DCA and LCA are largely recognized as genotoxic agents contributing to colon tumorigenesis. Indeed, DCA and LCA are capable of damaging DNA through oxidative and nitrosative attack, resulting in a higher rate of genetic instability [82]. Again, unhealthy diet habits are involved in DCA and LCA carcinogenesis since high fat consumption stimulates the production and secretion of bile acids and their conversion into elevated levels of genotoxic secondary derivatives [83].

Enteric commensal bacteria and pathogens may finally exert direct carcinogenic effects through the generation of genotoxic metabolites or bacterial genotoxins. Some pathobionts such as certain *E. coli* in group B2 produce the genotoxic colibactin. Colibactins (Clb) are secondary polyketide peptide metabolites produced by an enzymatic complex that comprises a total of eight non-ribosomal peptide synthetases (NPRSs) and polyketide synthases (PKSs). A total of 19 genes—collectively known as pks island—encode for different NPRSs, PKSs, and eight tailoring and editing enzymes, which through their assembly in enzymatic complexes synthesize the different Clbs (B, C, I, J, K, N, O) [84,85]. The pks island has been identified in *E. coli* strains and in other pathogens such as *Klebsiella* aerogenes and *Citrobacter koseri* found in gut microbiota or isolated from infectious disease and CRC cases [85]. Clbs are synthesized in the bacteria cytoplasm in the form of the non-genotoxic precursor pro-colibactin; pro-colibactin is transferred to the periplasmic compartment, where it is converted into the active Clbs [86]. The mechanism responsible for the transport of Clbs to the target cells’ nucleus is still unclear, but it requires microbial adhesion or invasion of host cells [84].

Clbs hits eukaryotic cells’ nuclei alkylating DNA and then inducing inter-strand DNA cross-links, subsequently converted into DNA double-strand breaks (DSBs): these lesions trigger cell-cycle arrest and foster CRC. DNA interstrand crosslinks and DSBs are particularly hard to repair and, if not accurately repaired, lead to DNA mutations and genomic instability, an observation that underpins the potential role of Clbs as CRC mediators [87]. Notably, DNA DSBs can be found in the epithelial cells of more than half of inflammatory bowel diseases and CRC patients [88]; Clb-DNA adducts have been detected in human and animal cells and in animal models [89]; inoculation with pks+ *E. coli* NC101 increases tumor formation and enables metastasis in mice [90]. This tumor-promoting activity is diminished with the deletion of the Clb gene cluster. Clbs synthesis inhibitors capable of blocking the effects of these genotoxins are being studied and developed. Their availability could have an important impact as a preventive strategy to impede Clbs production, and could provide a better understanding of the actual role of Clbs in CRC carcinogenesis [91].

Cytolethal distending toxins (CDTs) are another type of bacterial genotoxins discovered in *Campylobacter* spp and produced by more than 30 Gram species including *E. coli* and *Shigella*. CDT is a heterotrimeric complex mostly consisting of CdtA, CdtB, and CdtC subunits. CdtA and CdtC are regulatory subunits; the CdtB catalytic subunit possesses phosphatase and DNase activities, the latter causing DNA breakage [92]. Typhoid toxin secreted by *Salmonella tiphy*, although possessing a different structure as compared to CDTs, also contains the genotoxic CdtB subunit [93]. 

A thorough analysis of the kinetics and type of DNA damage caused by the *E. coli* CDT-I [94] revealed the presence of DNA single strand breaks (SSBs) within 3-6 h post-intoxication with low toxin doses (50 pg/mL); SSBs are then converted into DSBs during the S-phase as a consequence of the failed attempt to repair the former. Primary DSBs were instead reported after intoxication with high toxin doses (above 75 ng/mL) in a cell-cycle independent fashion, as a result of the presence of SSBs facing each other on the opposite strands [94,95]. 

It is of note that the patients diagnosed with severe infections sustained by Salmonella-produced CDT, particularly *S. enteritidis*, have an increased risk of developing cancer in the ascending/transverse parts of the colon [96]. Given the high worldwide number of infections caused by this major foodborne pathogen, a wide population-based cohort study was recently conducted in Denmark using four health registries from 1994 to 2016 in search of an association between laboratory-confirmed non-typhoidal salmonellosis and CRC diagnoses [97]. This wide epidemiological study did not confirm the results of the former [96] as no correlation was found between notified Salmonella infections and increased CRC incidence. An explanation for these apparently contradictory results has not been provided.

Finally, the highly toxic and proinflammatory ribosome-inactivating toxin Shiga toxin 1 (Stx1)—produced by *Shigella dysenteriae* and some *E. coli* serotypes responsible for bacillary dysentery, enterohemorrhagic colitis, and hemolytic uremic syndrome—has been shown to possess direct and articulated genotoxic activities. Human umbilical vein endothelial cells (HUVEC) intoxicated with very low concentrations of Stx1 (10 pM) accumulate significant levels of DNA SSBs resulting from the conversion of apurinic sites [98], a mechanism consistent with the enzymatic capacity of Stx1 to cleave adenine from nucleic acids in vitro. In addition to its DNA-damaging activity, Stx1 has also been shown to act as a DNA repair inhibitor at even lower (2.5–5 pM) concentrations [99] Under these conditions, Stx1 retarded the repair of oxidative and alkylative DNA lesions generated by H_2_O_2_ or methyl-methane-sulphonate in HUVEC cells. The inhibition of DNA repair has been hypothesized to depend on the precocious enzymatic cleavage of adenines from poly (ADPribose), the polymer involved in the post-translational regulation of DNA repair enzymes [99]. This is the unique report of the capacity of a bacterial toxin to damage DNA and to impair at the same time the DNA repair machinery of mammalian cells. This double interaction suggests that in the course of infections by *S. dysenteriae* or the above *E. coli* serotypes, Stx1 could directly damage DNA of intestinal epithelial cells and inhibit the repair of the DNA lesions produced by the genotoxic species normally released in inflamed gut; these DNA lesions could persist and be improperly repaired, thus augmenting their carcinogenic potential. Notably, the susceptibility to the infections from the above pathogens and their severity seem to be affected by basal gut microbiota composition [100]. Although no investigation has been addressed to see whether these infections effectively correlate with an increased risk of CRC, the above challenging hypothesis emphasizes the complicated and elusive interactions that may link gut microbiota to the onset of CRC.

## 4. Bidirectional Interactions between Drugs and Microbiota

Today a large variety of orally administered drugs are known to undergo gut microbiota biotransformation within the intestine and to affect microbial community composition, leading to the formation of metabolites with altered pharmacological profiles. These metabolic interactions may significantly contribute to the final pharmacokinetic and pharmacodynamic profiles of the parental drug [101,102] in such a way that the new term “pharmacomicrobiomics” was coined. The type and extent of these interactions may also vary as a function of the inter-individual differences of gut-microbiota composition, which may further and temporarily vary depending on extrinsic factors, concomitant antibiotic therapies above all. In this complex and not easily predictable scenario, the production of toxicologically relevant byproducts is also gaining increasing attention since teratogenic, genotoxic, and toxic microbial-derived metabolites have been identified [103,104].

In the opposite direction, increasing evidence indicates that the ability of modifying the composition of microbiota is not limited to antibiotics, but is shared by other widely used drugs used for the treatment of non-communicable diseases.

### Anticancer Drugs

The efficacy, the outcomes, and the severity of adverse effects of chemotherapeutic regimens can significantly vary between individuals bearing the same type of tumor. Similarly, to other classes of drugs, emerging evidence suggests that differences in the gut microbiota may concur to this phenomenon. In the case of cancer chemotherapy, microbiota may be relevant at either direct or indirect levels in that it can modify drugs’ chemical structures affecting their pharmacokinetic/dynamic fate and it is involved in the modulation of the host immune system. A recent study in which the cytotoxic activity toward various tumor cell lines of 30 anticancer drugs co-incubated with *E. coli* or *Listeria welshimeri* was determined, showed significant variations (increase or decrease) in the cytocidal activity in half of the tested drugs [105]. Further experiments with a selected panel of these drugs (gemcitabine, fludarabine, and CB1954) showed that *E. coli* exposure was capable of directly modifying their chemical structures. In the case of gemcitabine, it has been hypothesized that *E. coli* inactivates the drug through the acetylation of the amine group on the cytosine base. Notably, intratumor injection of *E. coli* (in an in vivo murine subcutaneous tumor model) reduced the efficacy of gemcitabine in a manner consistent with the observed metabolic activities [105]. 

In a different direction, severe diarrhea represents one of the major limitations to the use of the potent antitumor prodrug irinotecan, which is converted into its active derivative SN38 by carboxylesterases. In the liver, SN38 is inactivated via glucuronidation and then excreted through the bile. Once in the intestine, β-glucuronidases of the symbiotic bacteria remove the glucuronide, reactivating SN-38, which then causes diarrhea [106]. To block this complex circuit without killing the bacteria, specific inhibitors of the offending bacterial β-glucuronidases were selected and one of them was found to prevent irinotecan-induced diarrhea in mice [104].

Along with gemcitabine and irinotecan, the therapeutic efficacy of other widely used antitumor agents (5-fluorouracil, cyclophosphamide, oxaliplatin, methotrexate) has been shown to be intimately linked to interactions with host gut microbiota. Treatment using 5-fluorouracil results in a significantly altered gut microbiota, which may be responsible for its adverse effects including intestinal mucositis [107]. The microbial dysbiosis induced by 5-fluorouracil administration led to an increase in *F. nucleatum* and *Prevotella oris*, and a decrease in commensals from the genera *Streptococcus*, *Actinomyces* and *Veillonella. F. nucleatum* promoted CRC resistance to chemotherapy by orchestrating Toll-like receptors 4-Myeloid differentiation factor 88 (TLR4-MYD88) innate immune signaling pathway, specific miRNAs (genomic loss of miR-18a* and miR-4802) and autophagy elements (ULK1/ATG7 autophagy network) [108]. The administration of antibiotics reduced the efficacy of 5-fluorouracil, while the supplementation of *Bifidobacterium* and *Lactobacillus* strains could not improve the efficacy [109].

Microbial metabolites, particularly butyrate, are able to increase the oxaliplatin chemotherapeutic efficacy by regulating CD8+ T cell function, which plays a central role in tumor immunity [110]. In fact, responsive cancer subjects to oxaliplatin showed a higher abundance of serum butyrate compared to non-responding patients. Moreover, *B. fragilis* and other species improve intestinal immune reactions, enhancing the transcription of genes involved in immunity and lymphocytes’ infiltration, and lead to an improved effectiveness of oxaliplatin-chemotherapy [111].

Oxaliplatin causes a mechanical hyperalgesia in mice models, which can be reduced in mice pretreated with antibiotics and in germ-free mice. Restoring the microbiota of germ-free mice abrogated this protection. These effects appear to be mediated, in part, by TLR4 expressed on hematopoietic cells, including macrophages [112].

Recently, it has been shown that the integrity of microbiota plays a critical role in the clinical efficacy of the anti-programmed cell death (PD-) or anti-PD-ligand-1 (PD-L1) immune checkpoint inhibitors [113]. In particular, the study analyzed the influence of antibiotic drugs on the clinical outcomes of metastatic renal cell carcinoma patients treated with nivolumab or the combination of nivolumab and ipilimumab. The use of antibiotics significantly reduced the median progression free survival (2.8 months in patients treated with antibiotics and 18.4 months in patients not treated with antibiotics, respectively). 

From the toxicological point of view, 18 acute deaths in Japanese patients with cancer and herpes zoster have been ascribed to the negative interactions between a byproduct of gut-microbial metabolism of the antiviral drug sorivudine and the antitumor 5-fluorouracil prodrug Tegafur [114]. Under these co-administration conditions, very high levels of 5-fluorouracil accumulated in the blood of the patients, causing their death.

Microbiota is known to modulate the toxicity of the anticancer anthracycline doxorubicin at the intestinal level. In particular, the gut microbiota seems to promote protection toward the colonic epithelium and damage toward the small intestine [115]. It has been shown that, while in the colonic mucosa, commensal microbiota sustains epithelial integrity or promotes mucosal repair [115], in the small intestine it seems to exacerbate damage increasing epithelial permeability, which would in turn result in a detrimental inflammatory response. More recently, this differential effect has been explained based on the distinct bacterial metabolic routes involved in doxorubicin biotransformation. In particular, it has been shown that *Raoultella planticola* inactivates doxorubicin to 7-deoxydoxorubicinol and 7-deoxydoxorubicinolone via a reductive deglycosylation mechanism pathway based on molybdopterin-dependent enzyme. This reaction occurs only under the anaerobic conditions typical of the colon [116]. *Klebsiella pneumoniae* and specific strains of *E. coli* have been shown to drive a similar anaerobic detoxification route(s) [116]. On the other hand, although it has been observed only in environmental bacteria and not in intestinal species, under aerobic conditions similar to those found in the small intestine, doxorubicin bacterial metabolism involves an oxidative phosphorylation pathway leading to the production of the anthracycline aglycone semiquinone. The semiquinone autoxidizes and forms highly toxic ROS [117].

Overall, these observations suggest that specific microbiota functions might be targeted and/or preserved in the clinical practice to improve the efficacy, reduce the interindividual responses, and prevent the toxic burden of antitumor chemotherapeutic drugs.

Finally, the gut microbiome can indirectly impact the individual’s response to immunotherapy in cancer treatment via its influence on the host’s general immune status [118]. In particular, some anaerobic commensal bacteria, which number increases after dietary fiber intake, can enhance efficacy to checkpoint blockade immunotherapies. Moreover, fecal microbiota transplantation could be useful to enhance the clinical response in non-responding patients to those therapies.

Immunotherapy can impact on the gut microbiome, causing more complications. Dubin et al. demonstrated that the immunologic checkpoint blockade with ipilimumab is related to immune-mediated colitis. Patients with metastatic melanoma who undergo ipilimumab treatment develop colitis possess fewer bacteria from the Bacteroidetes phylum and also lower expression of genes involved in polyamine transport and B vitamin biosynthesis. This finding could lead to specific microbial interventions to reduce the risk of inflammatory complications following cancer immunotherapy [119].

## 5. Effects of Local Microbiota on Tumor Microenvironment

The dynamic interaction between cancer cells and tumor microenvironment (TME) affects cancer growth, invasion, metastasis, treatment resistance, and failure of immune defense systems. According to some estimates, microbial infections may be responsible for 20% of human malignancies [120]. The greatest proof that the microbiota is not only a bystander in the development of cancer is probably the *Helicobacter pylori*-induced gastritis and stomach adenocarcinoma [121], which are caused by carcinogenic microorganisms. Viral involvement in tumorigenesis is comparatively better understood [122].

As part of the intratumoral environment, local microbiota, which is tissue-, patient- and tumor-type-specific, can greatly influence prognosis and therapeutic outcome. A growing body of research indicates that local microbiota has a substantial role in the development of pancreatic, colon, lung, breast, and other cancers [123].

At a systemic level, the intestinal epithelial barrier’s hematopoietic and non-hematopoietic components can be modified by the gut microbiome, which can also stimulate the primary and secondary lymphoid organs and, in turn, control TME immune activity. The immune oncology microbiome (IOM) axis represents these immune-mediated interactions and collective feedback loops [124]. At a local level, the tumor immune microenvironment, including tissutal microbiota, can either accelerate or stop the development of cancer [125] depending on the type of cells and the TME signals. Dysbiosis in local bacterial populations can result in a persistent, pro-inflammatory immune response, which in turn promotes the development of cancer. For example, a microbial activation of NF-κB, a crucial regulator of cancer-related inflammation, has been demonstrated. Local microbiota can also inhibit the antitumor immune response [126].

The interaction between microorganisms and immunity is thought to be explained by several processes [127]. Microbial antigens can imitate tumor antigens and immunological cells may cause an immune response that results in effector T cells recognizing and killing the antigen-presenting cells. T lymphocytes can identify tumor cells that exhibit related antigen epitopes and, as a result, destroy them [128].

Microbes can interact with different pattern recognition receptors, with stimulatory or inhibitory finality, driving to a final response mediated by signaling pathways [129]. On the surface of tumor cells, there are receptors of metabolites such as short-chain fatty acids (SCFAs) produced by microbiota, suggesting that they can be potential regulators in the microenvironment [127]. Recent studies observed an interaction between specific bacteria and immune checkpoints that are important keys to regulating the immune cells [130].

Between normal and pathological paired tissues, there were reported to be significant variations in the amount and kind of bacteria [131]. Depending on the type and subtype of cancer, the intratumor microbiome varies. The percentage of tumors that contain bacterial genetic material varies depending on the type of tumor; for instance, melanoma has a positive rate of about 15%, while breast cancer has a higher positive rate of up to 60% [132].

The involvement of commensal bacteria in CRC has been proven in numerous studies. For example, *F. nucleatum* and its associated anaerobes are related with colorectal carcinoma [133]. *F. nucleatum* has been demonstrated to enhance chemoresistance downregulating microRNAs involved in the autophagy pathway [108]. On the other side, Shi et al. discovered that colonic *Bifidobacterium* accumulates in tumor locations and enhances local anti-CD47 treatment via the STING pathway [134].

Additionally, in esophageal cancer, a link between *F. nucleatum* presence in tumoral tissues and a bad prognosis has been demonstrated by Yamamura et al. suggesting a potential role as a prognostics biomarker since it might contribute to cancer aggressiveness through activation of chemokines [135].

Pancreatic cancer tissues contain intratumor microorganisms, and Geller and colleagues [4] discovered that Gammaproteobacteria were the predominant taxon in the human pancreatic ductal adenocarcinoma (PDAC) samples and might contribute to drug resistance and growth of pancreatic cancer cells.

The microbiome of malignant pancreases is substantially richer than that of healthy pancreases in both mice and humans, and several bacteria were shown to be more prevalent there than in the intestine. Furthermore, bacterial ablation protects against pre-invasive and invasive PDAC, whereas transfer of bacteria from tumor-bearing hosts, but not controls, reverses tumor protection [136]. Riquelme and colleagues [137] analyzed the tumor microbiome composition in PDAC patients with short-term survival (STS) and long-term survival (LTS). They observed a higher alpha-diversity in the tumor microbiome in the last one, and identified an intra-tumoral microbiome signature (*Pseudoxanthomonas-Streptomyces-Saccharopolyspora-Bacillus clausii*) highly predictive of long-term survivorship. The intestinal microbiota appears to control which bacteria can be identified in the tumor in the mouse model of pancreatic cancer [137]. This indicates that bacteria may migrate to the tumor at a later stage, when molecular and physical barriers may be destroyed, and there may be a relative immunosuppression, which may enhance the possibility of bacterial migration. Furthermore, all tumor-related bacteria are intracellular, suggesting the possibility that bacteria are carried by immune or cancer cell migration as whole or fragmented cells [137].

The lung is a site with a large exchange surface with the outside, constantly exposed to contact with microbes, with its own microbiota. The most abundant genera in healthy lung are *Prevotella*, *Streptococcus*, *Veillonella*, *Neisseria*, *Haemophilus*, and *Fusobacterium* [138]. Dysbiosis has been detected in lung cancer. Advanced stage cancers and increased Thermus genus abundance, as well as *Legionella* and metastases, are all related, and lung cancer patients’ tumor tissues possess low alpha diversity [138]. By stimulating lung-resident T cells, the lung cancer microbiome can contribute to the inflammation that accompanies lung adenocarcinoma. Certain bacteria can result in epithelial TP53 mutations in lung cancer tissues with squamous cell carcinoma [139]. Under both antibiotic and probiotic circumstances, tumor metastases were observed to be decreased and immunosuppression was overcome by the probiotic *Lactobacillus rhamnosus* [140].

Thompson et al. analyzed the microbiota in breast tissues and discovered diverse bacteria, more *Proteobacteria* in tumor samples and *Actinobacteria* in normal tissues [141]. Furthermore, *Methylobacterium radiotolerans* was discovered to be significantly common in breast tumor tissue while *Sphingomonas yanoikuyae* was shown to be more prevalent in paired normal tissue, where it can trigger defensive processes [131]. *F. nucleatum* DNA is prevalent also in breast cancer (BC) cells and hastens the development of metastatic BC blocking antitumor immunity [131]. The progesterone and testosterone-metabolizing enzyme 5-steroid hydrogenase is activated by *Bacillus cereus*, which has a distinct pro-carcinogenic impact [131]. Healthy tissues contain higher concentrations of *Lactococcus* and *Streptococcus* than BC tissues since they stimulate cellular immunity [142] and antioxidant protection [143]. In more recent studies, the local microbiota of BC patients with distinct molecular types (luminal A, luminal B, HER2-positive, or triple negative BC) was compared discovering distinctive bacterial fingerprints connected to each form of BC [144].

Changes in skin microbiome’s composition are linked to cancer, just like they are in other body regions. Melanoma samples were shown to be enriched in the *Fusobacterium* and *Trueperella* genera in a study of the microbiota of normal versus melanotic pig skin [145]. *Staphylococcus epidermidis*, a type of skin commensal bacterium producing 6-N-hydroxyaminopurine, was found to be protective against skin cancer in a cell culture investigation [146]. 

Intratumor bacteria differing from healthy tissue ones have been detected also for other cancer forms, suggesting not only an important role of local microbiota in tumorigenesis, but also the potential use for new preventive and therapeutic strategies.

## 6. Conclusions

Complex, interdependent mechanisms along with situational variables govern the opposite effect of microbiota on the onset and fate of human tumors, the balance between healthy and unhealthy microbiota being particularly critical. Data from literature are progressively increasing emphasizing the importance of this issue in cancer epidemiology, prevention, and therapy. Given the accumulating and mounting body of data from basic and clinical research, the need for critical and periodical revision of literature is of outmost importance and should be encouraged. 

## Figures and Tables

**Table 1:** In vitro antigenotoxic activity of probiotics.

Probiotic(s)	Strain(s)	Experimental Protocol(s)	Effect(s)	Test(s)	Reference
*Lb. casei*	DN 114001	Incubation of bacterial cells (10^9^ cfu/mL) with **IQ** (25 μg/mL) for 24 h in MRS broth or for 168 h in phosphate buffer	↓ Comet tail (both co-incubations)	Comet assay	[21]
		Incubation of bacterial cells (10^9^ cfu/mL) with **MelQx** (25 μg/mL) or **PhIP** (25 μg/mL) for 24 h in MRS broth or for 168 h in phosphate buffer	↓ Comet tail (only co-incubation in PBS		
*Lb. rhamnosus*	231	Incubation of bacterial cells (8 × 10^11^ cfu/mL) with **MeIQx** (10 μg/mL) (time not indicated)	↓ Revertant colonies	Ames test with *S. typhimurium* TA98 and TA100 strains	[22]
*Lb. casei* *Lb. acidophilus* *Lb. delbrueckii* subsp. *bulgaricus* *Lb. rhamnosus* *Lb. plantarum*	17, unspecified 15, unspecified 24, unspecified 6, unspecified 3, unspecified	Incubation of bacterial cells (10^8^–10^9^ cells/mL) with **4-NQO** (0.1 mM) for 150 min	↓ IF_SOS_	SOS chromotest with *E. coli* PQ37	[23]
			↓ Tail moment	Comet assay on Caco-2 cells	
			↓ Revertant colonies	Ames test with *S. typhimurium* TA100 strain	
*Lb. plantarum* *Debaryomyces hansenii* *Wickerhamomyces anomalus* *Pichia fermentans* *Torulaspora delbrueckii* *Hanseniaspora uvarum* *Metschnikowiaaff fructicola* *Metschnikowia raukaufii* *Candida apicola* *Meyerozyma guilliermondii* *Saccharomyces boulardii*	05, 013, N14, C9O4, C9S2 21B, CF1 LAB1, LAB30, LAB32, LAB40, LAB49, LAB62 LT21, LT52, LT53, LT99, LT100, ATCC 14917^TM^ WCSF, 1IMC 510R^®^, IMC 513R^®^ LG2, LG15 LUL14, TO8 TO1, TO10 TO2, TO3 TO5 RIB1, RIB3 LAM3 UV10 PR1 Codex©	Incubation of bacterial or yeast cells (10^8^–10^9^ cells /mL) with **4-NQO** (0.1 mM) for 150 min	↓ IF_SOS_	SOS chromotest with *E. coli* PQ37	[24]
*Bacillus subtilis* *Bacillus megaterium* *Bacillus firmus* *Bacillus pumilus*	ATCC 9799, ATCC 6051, ATCC 23857, ATCC 33677 ATCC 99 ATCC 17060 ATCC 7065	Incubation of bacterial cells (10^8^–10^9^ cells /mL) with **4-NQO** (0.1 mM) for 150 min	↓ IF_SOS_	SOS chromotest with *E. coli* PQ37	[25]
*Lb. delbrueckii* subsp. *bulgaricus* *Lb. casei* *Lb. rhamnosus* *Lb. acidophilus* *Lb. plantarum* *Bifidobacterium bifidum*	V2Z2, V2Z3, V2Z4, V2Z5, V2Z6, V2Z8, V2Z9, V5Z5, V5Z6, V5Z7, V5Z11, V2c, V5X5, 6a, 6b, 2b, 5a, 5b, 5c, 1a, 1b, J87, J88, J89 5H1, 5H2, 5H3, 5H4, 5H5, 5H6, 5H7, 5H8, 5H9, 5H10, C1, C2, C3, V5Z4, V5Z9, V5Z10, 2a J10, J30, J42, J54, J61, J62 J71, J72, J76, J77, A1, A2, A3, A4, A5, A7, A8, A9, A11, A43, A44 J1, J25, J40 J91, J92	Incubation of bacterial cells (10^8^–10^9^ cells /mL) with **4-NQO** (0.1 mM) for 150 min	↓ IF_SOS_	SOS chromotest with *E. coli* PQ37	[26]
*Lb. acidophilus* *Bifidobacterium bifidum* *Bifidobacterium infanti* *Bifidobacterium adolescentis* *Bifidobacterium breve* *Bifidobacterium longum*	2400, 2401, 2404, 2405, 2049, 2415 1900, 1901 1912 1920 1930 1941	Incubation of bacterial cells (concentration not indicated) with **NF**, **AMPIP**, **AMPI** (0.5 μg/plate) or **AFB1** (0.05 μg/plate) for 3 h	↓ Revertant colonies	Ames test with *S. typhimurium* TA98 and TA100 strains, with and without S9 mix as a metabolic activation system	[27]
*Lb. plantarum* subsp. *plantarum*	NIMBB003	Treatment of human lymphocytes with **AFB1** (10 μM) and bacterial cells (10^7^, 10^9^, 10^11^ cfu/mL)	↓ MN frequency	Micronucleus assay in human lymphocytes	[28]
*Lb. rhamnosus*	GG	Incubation of **AFB1** (150 μM) with bacterial cells (1 × 10^10^ and 5 × 10^10^) for 72 h	↓ DNA fragmentation induced by 25(OH)2D3	DNA fragmentation in Caco-2 cells	[29]
*Lb. rhamnosus* *Lb. brevis* *Lb. casei*	0908, 0900 0945 DN 114001, 0919	Treatment of HL60 cells with cell-free supernatants of bacteria (10^9^ cfu/mL; cultivated in MRS for 24 h or in phosphate buffer for 168 h) and **NMDA** (10 μg/mL) for 1 h	↓ Comet tail (both co-incubations)	Comet assay	[30]
*Streptococcus faecalis* subsp. *liquefaciens* *Streptococcus lactis* *Lb. casei* subsp. *casei* *Lb. casei* subsp. *rhamnosus* *Leuconostoc paramesenteroides* *Streptococcus lactis* subsp. *diacetylactis* *Streptococcus cremoris*	R-9, R-11, R-19, R-32, R-55 R-24 R-12, R-35, R-52, R-68 R-33 R-5, R-6, R-8, R-10, R-13, R-21, R-23, R-26, R-27, R-29, R-31, R-40, R-45, R-49, R-51, R-53, R-62, R-64 R-63, R-22, R-43 R-2, R-14, R-17, R-48	Incubation of lyophilized bacterial cells (5 mg) with **NDEA** (60 μM) for 1 h	↓ Revertant colonies	Ames test with *S. typhimurium* TA98 strain and S9 mix as a metabolic activation system	[31]
*Leuconostoc paramesenteroides* *Streptococcus lactis* subsp. *diacetylactis* *Streptococcus cremoris*	R-62, R-8 R-63 R-48	Incubation of lyophilized bacterial cells (3, 5, 7 mg) with **NMDA** (60 μM), **NPYR** (50 μM), or **NPIP** (50 μM) for 1 h	↓ Revertant colonies (only for NMDA)		
*Lb. rhamnosus*	IMC501	Incubation of bacterial cells (10^9^ cells/mL) with **4-NQO** (0.1 mM) for 150 min	↓ IF_SOS_	SOS chromotest with *E. coli* PQ37	[32]
*Lb. casei*	MSA1, MSA24, MSA21, MSA13, ATCC 393^T^, MSA4, MSA23, MSA8, MSA15, MSA11, MSA10, MSA25, MSA12, MSA6, MSA22, MSA20, MSA17, MSA7, MSA19, MSA3, MSA9, MSA18, MSA16, MSA14, MSA5	Incubation of bacterial cells (10^5–^10^9^ cfu/mL) with **4-NQO** (0.1 mM)	↓ IF_SOS_	SOS chromotest with *E. coli* PQ37	[33]
*Lb. plantarum* *Lb. casei* *Lb. brevis*	Unspecified Unspecified Unspecified	Incubation of bacterial cells with **NF** (concentrations and time were not indicated)	↓ Revertant colonies	Ames test with *S. typhimurium* TA100 strain, with and without S9 mix as a metabolic activation system	[34]
*Lb. salivarius*	FDB89	Incubation of bacterial cells (10^8^–10^9^ cfu/mL) and **4-NQO** (20 mg/L) for 3 h	↓ IF_SOS_	SOS chromotest with *E. coli* PQ37	[35]
*Lb. plantarum*	301102	Incubation of fermented milk, prepared with *Lb. plantarum* 301102 or 301102S (mutant strain), or lyophilized exopolysaccharide (EPS) solutions (0.01, 0.1, and 1.0 mg/mL) with **Trp-P-1** (0.1 mg/mL) for 30 min	↓ Revertant colonies (only with *Lb. plantarum* 301102S or lyophilized EPS)	Ames test with *S. typhimurium* TA98 strain and S9 mix as a metabolic activation system	[36]
*Lb. casei* *Lb. plantarum* *Lb. rhamnosus* *Lb. brevis* *Lb.* spp	C33, 306, 32C, 364, 66C, H5, 357, 362, 369G, 349, 350, 365, 347, 410, 394, 408, 300, 342, 398, 88b, 455, 417, 447, 53Be, 400, 353, 88, 391, 13A 66B, 337, 371, 4Ab, 62B, 8A, 45A, 36D, 301, 303, 8c, 56, 329, 48Ab, 336, 366, ZAR61, 61B, 43, 19B 94, 442, 25B, 14A, 93, 85 7A, 38Db, 38D, 41, 39D sp. 434, sp. 432, sp. 428	Incubation of bacterial cells (10^5^–10^9^ cfu/mL) with **4-NQO** (0.1 mM) for 150 min	↓ IF_SOS_	SOS chromotest with *E. coli* PQ37	[37]
*Enterococcus faecium* *Bacillus coagulans* *Lb. plantarum* *Saccharomyces cerevisiae* *Pichia anamola* *Cryptococcus albidus*	AdF1, AdF2, AdF3, AdF11 AdF4	Incubation of bacterial or yeast cells (10^8^–10^9^ cells/mL) with **4-NQO** (0.1 mM) for 150 min	↓ IF_SOS_	SOS chromotest with *E. coli* PQ37	[38]
	AdF5, Adf6, Adf7, Adf9, Adf10 Sc04, Sc08, SC12, Sc20 Sc17 Sc18		↓ Revertant colonies	Ames test with *S. typhimurium* TA100 strain	
*Lb. rhamnosus* *Lb. casei*	0900, 0908 0919	Treatment of Caco-2 cells with bacterial cells (1 × 10^9^ cfu/mL) for 1 h prior to **IQ** or **PhiP** (50 μg/mL) exposure for 10 min	↓ Comet tail	Comet assay in Caco-2 cells	[39]
*Bifidobacterium animalis* subsp. *lactis*	BI-04	Treatment of Caco-2 cells with inactivated bacterial cells (about 5 × 10^8^ cfu/mL) and **BaP** (50 μM) for 4 h	↓ Comet length, ↓tail moment, ↓tail length, ↓ olive tail moment	Comet assay in Caco-2 cells	[40]
*Bacillus clausii* *Bacillus subtilis* *Bacillus lentus* *Bacillus pumilus* *Bacillus firmus* *Bacillus megaterium* *Bacillus* sp.	O⁄C, N⁄R, SIN, T, DSM 8716^T^, DSM 9783, DSM 2512 LPM, ATCC 6051^T^, ATCC 33677, ATCC 23857, ATCC 9799 E2, V4, ATCC 10841^T^ ATCC 7061^T^, ATCC 7065 ATCC 14575^T^, ATCC 17060 ATCC 14946 718	Incubation of bacterial cells (10^8^–10^9^ cells/mL) with **4-NQO** (0.1 mM), **AFB1** (0.05 mM) or **MeIQ** (0.16 mM) for 150 min	↓ IF_SOS_	SOS chromotest with *E. coli* PQ37, with or without S9 mix as a metabolic activation system	[41]
*Lb. acidophilus* *Lb. casei* *Lb. delbrueckii* subsp. *bulgaricus* *Lb. fermentum* *Lb*. *plantarum* *Lb. rhamnosus* *Bifidobacterium longum*	J76 5H10 J87 Unspecified J25 J54 Unspecified	Incubation of bacterial cells (10^8^–10^9^ cells/mL) with **aqueous extracts of heated oils** and their dilution (1:1 and 1:3) for 150 min	↓ IF_SOS_	SOS chromotest with *E. coli* PQ37	[42]
*E. coli*	Nissle 1917	Treatment of Caco-2 cells with bacterial cells (10^9^–10^10^ cfu/mL) and **4-NQO** (20 μg/mL) for 150 min or **BaP** (100 μg/mL) for 90 min	↓ Comet tail	Comet assay in Caco-2 cells	[43]
		Incubation of bacterial cells (10^9^ cfu/mL) with **4-NQO** (20 μg/mL) or **BaP** (100 μg/mL) for 20 min	↓ Revertant colonies	Ames test with *S. Typhimurium* TA100 and *E. coli* WP2 strains, with and without S9 mix as a metabolic activation system	

Abbreviations: ↓decrease; 4-NQO: 4-nitroquinoline 1-oxide; AFB1: aflatoxin B1; AMPI: 2-amino-3-methyl-9H-pyrido (3,3-6) indole; AMPIP: 2-amino-1-methyl-6-phenyl-imidazo (4,5-b) pyridine; BaP: benzo[*a*]pyrene; IQ: 2-amino-3-methyl imidazo[4,5-f]quinoline; MeIQ: 2-amino-3,4-dimethylimidazo(4,5-f)quinoline, 2-amino-3,4-dimethylomidazo[4,5-f]quinoline; MeIQx: 2-amino-3,8-dimethylimidazo[4,5-f]quinoxaline; NDEA: N-nitroso-diethylamine; NMDA: N-nitroso-dimethylamine; NF: 2-nitroflourene; NPIP: N-nitrosopiperidine; NPYR: N-nitrosopyrrolidine; PhIP: 2-amino-1-methyl-6-phenylimidazo[4,5-b]pyridine; Trp-P-1: 3-amino-1,4-dimethyl-5H-pyrido[4,3-b]indole. IF_SOS_: SOS induction factor, defined as the ratio between β-galactosidase to alkaline phosphatase (constitutive) of sample divided by the same ratio of negative control.

**Table 2:** In vivo antigenotoxic activity of probiotics.

Probiotic(s)	Experimental Protocol(s)	Experimental Model	Effect(s)	Test(s)	References
*Lb. rhamnosus* (IMC501)	Administration of bacterial cells (10^9^ cells/mL, 10 mL/kg b.w.) for 10 days before **PhIP** administration (100 mg/kg b.w.)	Male CD-1 mice	↓ Tail length	Comet assay on peripheral blood	[44]
*Streptococcus faecalis* (T-110) *Clostridium butyricum* (TO-A) *Bacillus mesentericus* (TO-A)	Administration of *Streptococcus faecalis* (10^8^ cfu/g), *Clostridium bothrium* (10^7^ cfu/g) and *Bacillus mesentericus* (10^6^ cfu/g) for 2 weeks before **AAC** (40 mg/kg b.w.) administration once a day for 3 days	HFA mice	↓ DNA adduct formation	^32^P-postlabelling assay on colonic epithelium	[45]
*Lb. plantarum* (CICC 23121)	Administration of bacterial cells (5 × 10^10^ cfu/mL) and **BaP** (50 mg/kg) twice a week for 28 days	Clean-grade Kunming mice	↓ Tail length	Comet assay on peripheral blood cells	[46]
Fermented milk (FM) supplemented with *Lb. rhamnosus* (GG) and *Lb. casei* (strain shirota)	(1) Administration of **AFB1** (450 μg/kg b.w) from week 4 to week 10 and of FM (10^8^ cfu/g) from week 4 to week 25 (2) Administration of **AFB1** (450 μg/kg b.w.) from week 4 to week 10 and of FM (10^8^ cfu/g) from week 10 to week 25 (3) Administration of **AFB1** (450 μg/kg b.w.) from week 4 to week 10 and of FM (10^8^ cfu/g) from week 1 to week 25	Male Wistar rats	↓ DNA damage scoring (cells with comet)	Comet assay on liver cells	[47]
*Saccharomyces cerevisiae* (unspecified strain)	Administration of yeast cells (10^8^ viable cells) and corn contaminated with **AFB1** (400 and 800 μg/kg) for 6 weeks	Male CD-1 mice	↓ Frequency of MNNE	Micronucleus test on mice erythrocytes	[48]
*Lb. rhamnosus* (GG)	Administration of bacterial cells (1 × 10^10^ cfu) 2h before **AFB1**, **AFB2**, **AFG1**, **AFG2** (0.7 mg/kg b.w.) administration every day for 7 consecutive days	Male Swiss Albino mice	↓ Structural and numerical chromosome aberrations, ↑ mitotic activity, ↑ meiotic activity	Micronucleus assay in mice bone marrow cells and in mice spermatocytes	[49]
*Saccharomyces cerevisiae* (RC016)	(1) Administration of **AFB1** (40 μg/kg) + **AFG1** (20 μg/kg) and yeast cells (10^8^ viable cells) daily for 60 days (2) Administration of **AFB1** (100 μg/kg) + **AFG1** (50 μg/kg) and yeast cells (10^8^ viable cells) daily for 60 days	Inbred male Wistar rats	↓ % MNPCE	Micronucleus test on rat erythrocytes	[50]
			No difference of tail moment	Comet assay on rat lymphocytes	
*Pediococcus acidilactici* (NNRL B-5627) *Lb. delbrueckii* subsp. *lactis* (DSM 20076)	Administration of bacterial cells (10^10^ cfu/mL) and **fumonisin B1** (100 and 200 mg/kg) once a day for 4 weeks	Sprague-Dawley rats	↓ DNA fragmentation	Analysis of DNA fragmentation on blood cells	[51]
*Lb. salivarius* (REN)	(1) Administration of bacterial cells (5 × 10^6^, 5 × 10^8^, or 5 × 10^10^ cfu/kg b.w.) once a day from week 1 to week 32 and **4-NQO** (20 ppm) from week 2 to week 9 (2) Administration of **4-NQO** (20 ppm) from week 2 to week 9 and bacterial cells (5 × 10^6^, 5 × 10^8^, or 5 × 10^10^ cfu/kg b.w.) once a day from week 10 to week 32	Male F344 rats	↓ 8-OHdG levels	Measurement of 8-OHdG levels on rat tongue epithelium	[52]

Abbreviations: ↓: decrease; MNPCE%: percentage of micronucleated polychromatic erythrocytes (MNPCE) over total erythrocytes; 4-NQO: 4-nitroquinoline 1-oxide; 8-OHdG: 8-hydroxydeoxyguanosine; AAC: 2-amino-alpha-carboline; AFB1: aflatoxin B1; AFB2: aflatoxin B2; AFG1: aflatoxin G1; AFG2: aflatoxin G2; BaP: benzo[a]pyrene; MNNE: micronucleated normochromatic erytrocytes.
